# Smokeless tobacco keratosis in oral mucosa with epithelial dysplasia: A case report

**DOI:** 10.1097/MD.0000000000037771

**Published:** 2024-04-12

**Authors:** Hassan Hamed Kaabi

**Affiliations:** a Assistant Professor, Department of Oral Medicine and Diagnostic Sciences, College of Dentistry, King Saud University, Riyadh, Saudi Arabia.

**Keywords:** case report, epithelial dysplasia, oral mucosa, smokeless tobacco keratosis, tobacco cessation

## Abstract

**Rationale:**

Smokeless tobacco use is a risk factor for the development of various oral lesions, among which is smokeless tobacco keratosis (STK). This condition is caused by constant frictional irritation of smokeless tobacco products against the oral mucosa and appears as a White-to-gray plaque with wrinkling.

**Patient concerns:**

A 50-year-old man who had been using smokeless tobacco for 24 years visited our clinic complaining of changes in the lower right sulcus of the oral cavity for 10 days. Clinical examination revealed a unilateral, nonhomogeneous White lesion in the area of the complaint. Histopathological examination showed hyperkeratosis, areas of keratin plugging, and mild dysplastic epithelial changes.

**Diagnosis:**

The clinico-histopathological correlation suggested a diagnosis of STK with focal mild epithelial dysplasia.

**Intervention and outcome:**

A comprehensive management plan included maintaining oral hygiene, education on the detrimental effects of smokeless tobacco, advice to cease smoking, and regular follow-up to monitor the potential for malignant transformation. The patient was referred to a tobacco cessation society for tailored advice and counseling. On follow-up visits, there was an improvement in the lesion after habitual cessation.

**Lessons:**

The diagnosis of tobacco-related oral lesions is often delayed, which may result in malignant transformation. This illustrates the need to train healthcare professionals to identify tobacco-related conditions at an early stage and to educate patients regarding the harmful effects of tobacco use.

## 1. Introduction

White lesions in the oral mucosa are commonly encountered in routine dental practices. Diagnosis of these lesions may be challenging for dental professionals. Several White oral lesions have the potential to transform into malignancies, for which early diagnosis is necessary to minimize progression to oral cancer.^[1]^

Smokeless tobacco use is a risk factor for the development of various oral White lesions, including smokeless tobacco keratosis (STK). This condition is caused by constant frictional irritation of smokeless tobacco products against the oral mucosa. The thickness of keratin formed depends on the frequency of habit and the amount used.^[2]^ STK appears as a White-to-gray plaque with slight wrinkling in its early stages. Advanced lesions represent excessive keratinization with clear pouching of the mucosa. Histologically, the epithelium is characterized by cyclic parakeratosis with signs of acanthosis. Epithelial dysplasia and hyperchromatic nuclei in the basal layer have been identified in several cases of STK. The connective tissue has minimal involvement, primarily collagen sclerosis around blood vessels and nerves.^[1]^ Most STK lesions usually disappear within a few weeks to months once the tobacco habit is discontinued; however, it is associated with a slightly increased risk of mouth cancer. Dental practitioners should educate patients about the potential harm caused by tobacco use and motivate them to stop smoking.^[1,3]^

This report describes a case of STK in a 50-year-old male patient. Ethical approval was obtained from the King Saud University Institutional Review Board (IRB no. E-23-8344). Complete written informed consent was obtained from the patient to use his data and images for publication.

## 2. Case report

A 50-year-old Indian male patient visited the Oral Medicine Clinics at King Saud University Dental Hospital in Riyadh, Saudi Arabia, complaining of changes in the sulcus of the lower right side for 10 days. The patient did not experience any pain, burning sensations, or dysgeusia. His habit history included the use of smokeless tobacco for 24 years. He keeps the tobacco (moist snuff, Afzal) in the lower right vestibule of his jaw for 10 hours per day and then spits it out. The interview revealed that he had no significant medical history and had not taken any medication or consumed alcohol.

**Figure 1.**
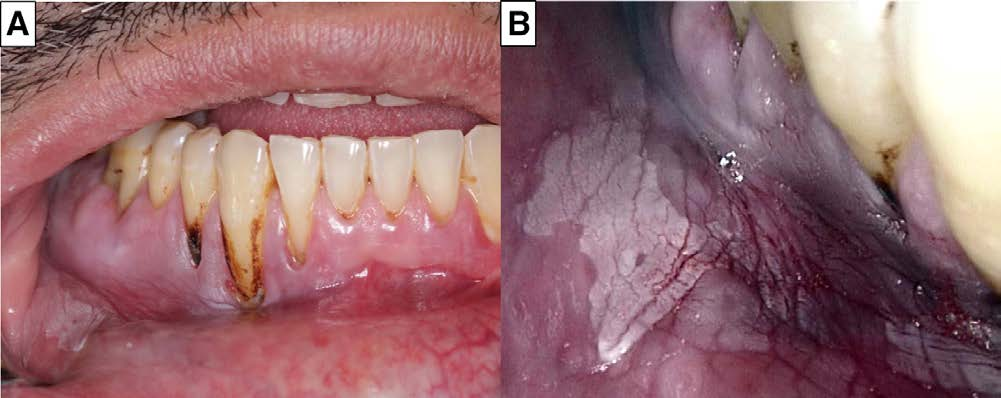
Clinical photographs. (A and B) Unilateral non-homogeneous White lesion in the lower right buccal sulcus associated with gingival recession and teeth staining.

An extraoral examination revealed no abnormalities in the temporomandibular joint, salivary glands, or masticatory muscles. Intraoral examination showed a unilateral nonhomogeneous red and White lesion in the lower right buccal sulcus and gingival margins extending from the canine to the first molar (measuring 1.8 cm × 1.1 cm). Gingival recession and tobacco staining are evident on the lower right side. His overall oral hygiene was poor. On palpation, the lesion had a soft, rubbery texture. Clinical photographs of patients were obtained (Fig. 1). Upon review of the patient’s history and comprehensive examination, a diagnosis of non-homogenous leukoplakia was made, and a biopsy was performed.

Under topical and local anesthesia, a representative strip of soft tissue biopsy (0.7 cm × 0.5 cm × 0.4 cm) was taken from the lower buccal right vestibule area of teeth #43 to #46 and placed in 10% buffered formalin for fixation. Bleeding was managed with single interrupted sutures, and the patient was given postoperative instructions.

**Figure 2.**
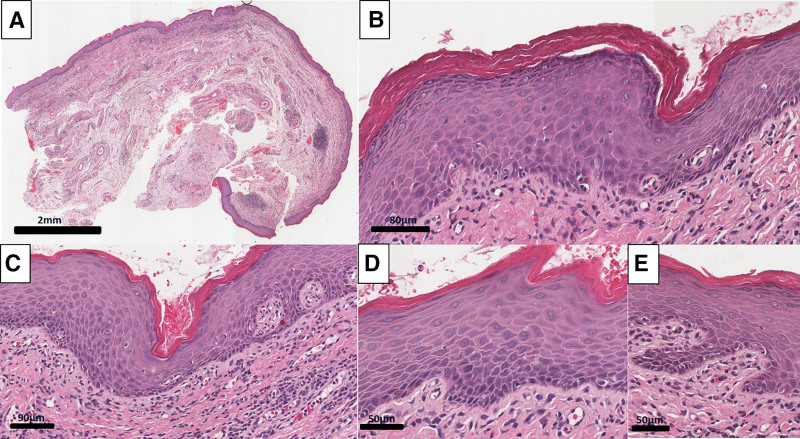
H&E histopathology of biopsy from the lower buccal right vestibule area. (A) low magnification; (B) hyperkeratosis; (C) keratin plugging; (D and E) mild dysplastic changes in the basal and parabasal layers.

The specimen was ground by an oral pathologist, processed, embedded in paraffin wax, and stained with hematoxylin and eosin. The stratified squamous epithelial tissue exhibited hyperkeratosis, areas of keratin plugging, and mild focal dysplastic changes in the basal and parabasal layers (Fig. 2). Accordingly, the clinico-pathological correlation suggested a diagnosis of STK and mild focal epithelial dysplasia of the right buccal sulcus.

Scaling and using mouthwash to maintain oral hygiene were recommended as part of the comprehensive treatment plan. The patient was educated about the detrimental effects of using smokeless tobacco and advised to cease the habit to help improve the lesion. Regular follow-up was recommended, and the patient was informed of the possibility of requiring an additional biopsy if any suspicious areas were observed. The patient was referred to a tobacco cessation society for tailored advice and counseling.

**Figure 3.**
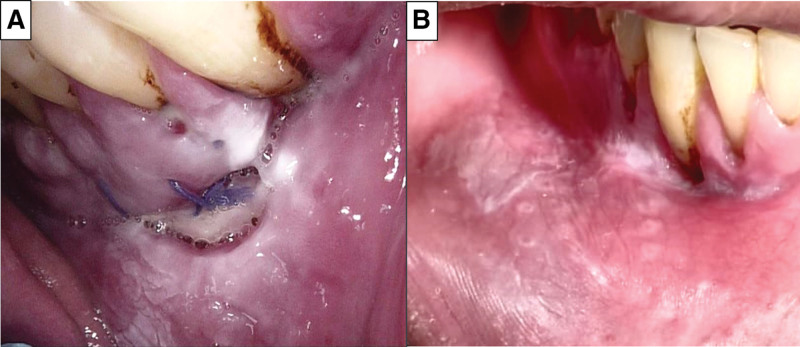
Clinical photographs. (A) 1 wk, and (B) 2 mo post-biopsy showing soft tissue healing, with no sign of malignancy.

One week after the biopsy, the patient underwent suture removal and follow-up. He experienced mild pain and edema at the biopsy site with no bleeding. The soft tissue in the incisional area healed with no signs suggestive of malignancy (Fig. 3A). On the 2-month follow-up visit, there was significant improvement in lesion extension, morphology, and homogeneity after habitual cessation (Fig. 3B). However, the patient was informed that if the lesion persists or progresses, complete removal will be performed.

## 3. Discussion

A key issue in the context of oral lesions is the delay in diagnosis. The patient in this report was diagnosed with STK 24 years after smokeless tobacco use. STK usually develops within 1 to 5 years of tobacco use and has the potential to transform into malignancy if the habit is not stopped.^[2,4]^ The delay in diagnosis is attributed to various factors, including the level of knowledge and competencies of dental care providers and the lack of awareness among patients. There is a low level of knowledge about oral cancer among patients relative to other types of cancer. Therefore, they reported the condition in the late stages, which required a more invasive treatment plan. Interventions to improve public knowledge of oral cancer and the referral of high-risk patients with oral cancer (such as heavy tobacco users) to specialists could result in early diagnosis and management.^[5–7]^

Tobacco use is a critical public health issue and remains a significant cause of premature death worldwide. The patterns of tobacco use include conventional cigarettes, smokeless tobacco, waterpipes, cigars, and e-cigarettes.^[8]^ Smokeless tobacco includes unburned products that can be used orally or nasally, including chewing, snus, dry snuff, and dissolvable tobacco.^[2,9]^ It is estimated that 21.4% of adults regularly consume smokeless tobacco products, which is twice that of smoking, most of which are from developing countries. There is also a widespread use of smokeless tobacco among adolescents and children.^[10]^ Aside from nicotine, which is highly addictive, smokeless tobacco also contains many carcinogenic compounds that can lead to a range of health problems.^[11]^ High doses and frequency may worsen the case and increase the risk of malignancy transformation.^[1,12]^ A variety of oral effects can be associated with the use of smokeless tobacco, including oral cancer, erythroplakia, leukoplakia, submucous fibrosis, periodontal disease, and staining of the teeth.^[13]^

STK was found to be the most common lesion among smokeless tobacco users.^[14]^ It develops in 60% of smokeless tobacco users.^[15]^ STK is generally described as a benign mucosal White lesion located where the smokeless tobacco is held in the oral cavity, primarily the buccal sulcus. There is a high prevalence of gingival recession and periodontal disease among smokeless tobacco users.^[2]^ Early STK lesions are characterized by White films in the mucosa. Over time, chronic irritation results in excessive keratin deposition on the top layer of the epithelium, which causes a White-corrugated appearance of the mucosa. STK is a risk factor for oral cancer, particularly when the lesion is associated with epithelial dysplasia. The histopathological features of STK lesions were not specific. The superficial and spinous layers of the epithelium exhibit hyperkeratosis and acanthosis, respectively. Cell dysplasia in the basal epithelial layer and collagen sclerosis in the lamina propria are reported in a few cases.^[1]^

STK can be diagnosed by patient interviews and thorough examination of the oral mucosa. Wrinkled White mucosal lesions, which correlate with the use of smokeless tobacco products, may be used as indicators of STK. The presence of ulceration, change in color from White to red, and enlargement of the mucosal lesion may require biopsy and further monitoring for potential malignant transformation. It is prudent to carefully examine biopsies for malignant changes, as the level of epithelial dysplasia may guide the choice of surgical intervention or monitoring. The following conditions may show clinical features similar to those of STK: oral leukoplakia, frictional keratosis, and oral squamous cell carcinoma.^[1,15,16]^ In most cases, the oral mucosa is restored within 2 to 6 weeks of cessation of the habit. However, the lesion cannot be resolved if this habit is not stopped, the lesion cannot be resolved. Regular follow-up is advised, and the patient should be informed about the possibility of the need for further biopsy if any suspicious signs of malignancy are observed.^[1,3]^

It is essential to have an effective healthcare system to reduce the demand for tobacco and to offer cessation treatment and support. Clinical practice guidelines for tobacco treatment recommend that healthcare providers implement the 5As and 5Rs models. The 5As model (Ask, Advise, Assess, Assist, and Arrange) helps tobacco users attempt to quit. In contrast, the 5Rs model (Relevance, Risks, Rewards, Roadblocks, Repetition) motivates tobacco users who are unwilling to stop.^[17]^ At the local level, the Ministry of Health of Saudi Arabia has developed fixed and mobile tobacco cessation clinics distributed nationwide. These clinics offer various services, including medical consultations, behavioral therapy, pharmaceutical medications, and follow-up support. The success of tobacco cessation depends mainly on the public’s awareness of these initiatives and utilization of tobacco cessation services.^[8]^

## 4. Conclusion

STK is a benign oral mucosal lesion with potential for malignant transformation in response to smokeless tobacco use. Clinical diagnosis of STK may be difficult; therefore, a biopsy can be performed to eliminate the possibility of malignancy. There is usually a resolution of STK following cessation of tobacco use; however, if the lesion persists or dysplasia is seen on histology, it may need to be monitored periodically or removed surgically. The role of healthcare professionals in the early identification of tobacco-related conditions is crucial, and patients must be educated on the harmful consequences associated with the chronic use of smokeless tobacco. It is imperative to screen patients for tobacco use and to offer brief cessation interventions to all tobacco users.

## Acknowledgments

The author would like to thank Dr Abdullah M. Alsoghier (College of Dentistry, King Saud University) for his great assistance in preparing the manuscript.

## Author contributions

**Conceptualization:** Hassan Hamed Kaabi.

**Data curation:** Hassan Hamed Kaabi.

**Investigation:** Hassan Hamed Kaabi.

**Writing – original draft:** Hassan Hamed Kaabi.

**Writing – review & editing:** Hassan Hamed Kaabi.

## References

[1] Müller S. Frictional keratosis, contact keratosis and smokeless tobacco keratosis: features of reactive white lesions of the oral mucosa. Head Neck Pathol. 2019;13:16–24. 30671762 10.1007/s12105-018-0986-3 PMC6405791

[2] Donald PM Renjith G Arora A. Tobacco pouch keratosis in a young individual: a brief description. J Indian Soc Periodontol. 2017;21:249–51. 29440796 10.4103/jisp.jisp_109_17 PMC5803885

[3] Binmadi N Harere L Mattar A. Oral lesions associated with smokeless tobacco users in Saudi Arabia: single center cross-sectional study. Saudi Dent J. 2022;34:114–20. 35241900 10.1016/j.sdentj.2021.12.002 PMC8864373

[4] Thakur DV Kaur DM Jassal DS. Clinical case report on smokeless tobacco keratosis. J Curr Med Res Opinion. 2020;3.

[5] Al-Maweri SA Al-Soneidar WA Dhaifullah E. Oral cancer: awareness and knowledge among dental patients in Riyadh. J Cancer Educ. 2017;32:308–13. 26423059 10.1007/s13187-015-0924-y

[6] Ford PJ Farah CS. Early detection and diagnosis of oral cancer: strategies for improvement. J Cancer Policy. 2013;1:e2–7.

[7] Jafer M Crutzen R Moafa I. What do dentists and dental students think of oral cancer and its control and prevention strategies? a qaualitative study in Jazan dental school. J Cancer Educ. 2021;36:134–42. 31506768 10.1007/s13187-019-01609-z PMC7835163

[8] Monshi SS Alanazi AMM Alzahrani AM. Awareness and utilization of smoking cessation clinics in Saudi Arabia, findings from the 2019 global adult tobacco survey. Subst Abuse Treat Prev Policy. 2023;18:33. 37322497 10.1186/s13011-023-00543-0 PMC10268372

[9] Hajat C Stein E Ramstrom L. The health impact of smokeless tobacco products: a systematic review. Harm Reduct J. 2021;18:123. 34863207 10.1186/s12954-021-00557-6 PMC8643012

[10] Bharati B Sahu KS Pati S. Prevalence of smokeless tobacco use in India and its association with various occupations: a LASI study. Front Public Health. 2023;11:1005103. 36923032 10.3389/fpubh.2023.1005103 PMC10008850

[11] Hecht SS Hatsukami DK. Smokeless tobacco and cigarette smoking: chemical mechanisms and cancer prevention. Nat Rev Cancer. 2022;22:143–55. 34980891 10.1038/s41568-021-00423-4 PMC9308447

[12] Greer RO Jr. Oral manifestations of smokeless tobacco use. Otolaryngol Clin North Am. 2011;44:31–56, v. 21093622 10.1016/j.otc.2010.09.002

[13] Muthukrishnan A Warnakulasuriya S. Oral health consequences of smokeless tobacco use. Indian J Med Res. 2018;148:35–40. 30264752 10.4103/ijmr.IJMR_1793_17 PMC6172921

[14] Choudhary A Kesarwani P Chakrabarty S. Prevalence of tobacco-associated oral mucosal lesion in Hazaribagh population: a cross-sectional study. J Family Med Prim Care. 2022;11:4705–10. 36352979 10.4103/jfmpc.jfmpc_1990_21 PMC9638602

[15] Petruzzelli CJ Varano A Desrosiers A. Smokeless tobacco keratosis. Dermatol Online J. 2023;29. 10.5070/D329361430 37591270

[16] Jones KB Jordan R. White lesions in the oral cavity: clinical presentation, diagnosis, and treatment. Semin Cutan Med Surg. 2015;34:161–70. 26650693 10.12788/j.sder.2015.0180

[17] Luo JG Han L Chen LW. Effect of intensive personalized “5As+5Rs” intervention on smoking cessation in hospitalized acute coronary syndrome patients not ready to quit immediately: a randomized controlled trial. Nicotine Tob Res. 2018;20:596–605. 28637193 10.1093/ntr/ntx126

